# Mediastinal Germ Cell Tumors: Up Close and Personal

**DOI:** 10.2196/93678

**Published:** 2026-03-20

**Authors:** B Wayne Bequette

**Affiliations:** 1 Department of Chemical and Biological Engineering, Rensselaer Polytechnic Institute, 110 Eighth Street, Troy, NY, 12180-3590, United States, 1 518-276-6683; 2 Center for Engineering and Precision Medicine, New York, NY, United States

**Keywords:** germ cell tumor, mediastinal tumors, rare cancers, cancer treatment, acute myeloid leukemia

## Abstract

Mediastinal germ cell tumors and how they differ from those associated with testicular cancer are reviewed through the experience with my son, Brendan Fahy Bequette. We reflect on others who have been diagnosed with rare tumors and discuss our need to honor Brendan’s memory by “paying it forward.”

## Background

Testicular cancer treatment is a great success story, largely due to the efforts of Dr Lawrence Einhorn at Indiana University to develop cisplatin-based chemotherapy protocols, with initial clinical trial success in 1974 [1,2]. Currently, someone diagnosed with testicular cancer has a 5-year expected survival rate of over 95%. A high-profile example is the cyclist Lance Armstrong, who was treated by Dr Einhorn in 1996. Germ cell tumors form the bulk of testicular cancers (95%). The TP53 gene is known as a tumor suppressor gene, and 50% of cancers have a TP53 gene mutation. Testicular cancers, however, have a low rate of TP53 mutations, contributing to the success of platinum-based chemotherapy treatment.

Mediastinal (in the chest between the lungs) germ cell tumors are very rare (2% of germ cell tumors) with a much lower treatment success than those that present in the testicles [3,4]. Unlike testicular cancers, mediastinal germ cell tumors tend to have a high rate of TP53 mutations. Nonseminoma mediastinal tumors treated with standard BEP (bleomycin, etoposide, cisplatin)–VIP (etoposide, ifosfamide, cisplatin) protocols have an approximately 5-year survival rate of 50%, which includes second-line treatment involving high dose chemotherapy with autologous stem cell transplant, where a patient’s stem cells are separated from their blood and reinfused as part of the treatment [5]. Unfortunately, a relapse after high-dose chemotherapy results in a median survival time of 5 months. There are ongoing efforts to develop immunotherapy [6] and cell and gene (chimeric antigen receptor [CAR-T]) treatments for patients who have relapsed (often called platinum-resistant).

## Personal Background

My son, Brendan Fahy Bequette, a filmmaker in New York City, was diagnosed with a mediastinal germ cell tumor in 2020 during the peak of the COVID-19 pandemic. While preparing for his initial treatment, we had a personal family contact with Dr Einhorn, who asked “how did Brendan know?” because a germ cell tumor normally progresses much further than Brendan’s before detection. Brendan was a runner and had noted a pain in his chest before going to an outpatient clinic and receiving a chest X-ray 2 days before his 24th birthday. Dr Einhorn immediately contacted Dr David Shaffer at Albany Medical College (AMC), who was providing the treatment. Dr Shaffer also began correspondence with Dr Darren Feldman at Memorial Sloan Kettering Cancer Center (MSKCC) in New York. Clearly in this field of rare cancers, the specialists know each other quite well.

Brendan received his first line of treatment, BEP-VIP, at AMC; unfortunately, a brain scan indicated a metastasis, and his second line of treatment involved high-dose chemotherapy with an autologous stem cell transplant at MSKCC. While in remission, he began training for a marathon as a way of celebrating his conquering of cancer; unfortunately, the cancer returned after 2 months.

His third line of treatment was in an immunotherapy clinical trial using durvalumab and tremelimumab. Unfortunately, the cancer progressed, and he was removed from the trial after 1 month. As often happens with immunotherapy, his immune system had responded by attacking his thyroid and affecting his heart rate, causing him to need thyroid medication for the rest of his life. Overall, the clinical trial failed to meet the protocol-defined success for the 29 patients treated [7]. For the next 6 months, Brendan received several other lines of treatment, including 4 months of cisplatin plus epirubicin.

Brendan’s last hope was a clinical trial for a novel CAR-T treatment for solid tumors [8]. Unfortunately, the trial was delayed by over 6 months while the sponsor automated the process, and Brendan died on February 28, 2022, two weeks after the last enrollment.

## Grief and Future Steps

My wife, Pat Fahy, and I have channeled our grief by publishing a book (The Photographs of Brendan Fahy Bequette) of black-and-white photos, many of which Brendan had taken during his cancer treatment [9,10]. The proceeds of this book support art and film awards in his name, thus “paying it forward” for young artists. When included with college scholarships in his memory, we honor approximately 8 young artists per year.

## Further Reflections

The New Yorker article by Tatiana Schlossberg (John F Kennedy’s granddaughter), “A Battle With My Blood,” is subtitled “When I was diagnosed with leukemia, my first thought was that this couldn’t be happening to me, to my family” [11].

Similarly, when we learned that our son had a tumor in his chest, Pat and I experienced the “this is not happening to us” state while simultaneously realizing that it was and that we needed to help Brendan start making a lot of important decisions about his health care. Tatiana goes on:

*I did not—could not—believe that they were talking about me. I had swum a mile in the pool the day before, nine months pregnant. I wasn’t sick. I didn’t feel sick. I was actually one of the healthiest people I knew. I regularly ran five to ten miles in Central Park.*

Brendan was also fit, having been a long-distance runner since his freshman year of high school. Indeed, the reason that he went to an outpatient clinic was that he could feel a pain near his heart when sleeping the previous night—he was seriously in touch with his body. As a filmmaker in New York, he also often ran in Central Park.

When Pat and I hear that someone has been diagnosed with cancer, we immediately wonder or ask, “what type of cancer?” Tatiana was diagnosed with acute myeloid leukemia (AML) with chromosome 3 inversion, which is rare (roughly 1.5% of AML cases). Similarly, Brendan was diagnosed with a mediastinal germ cell tumor, which is approximately 2% of all germ cell tumors.

Roughly 200 or so people are diagnosed with AML with chromosome 3 inversion in the United States each year. This is almost the same as the roughly 200 or so people diagnosed with a mediastinal germ cell tumor each year in the United States. Thus, both Tatiana and Brendan had rare cancers. Notably, both were treated at MSKCC.

## Role of Chemical Engineers in Therapeutics Manufacturing

Chemical engineers are largely responsible for the design and operation of the manufacturing plants that produce pharmaceuticals. Although the chemical engineering curriculum focuses on mathematical models and “algorithms,” I encourage my students to think about the “human in the loop,” which includes the safety of the personnel operating the plants and the consumer using the final product. I provide personal examples, such as my sister who was diagnosed with type 1 diabetes in 1977. This motivated me to collaborate on the development of an automated insulin delivery system to improve the lives of people living with type 1 diabetes. Similarly, I use the example of my son to motivate the ongoing development of immunotherapies for the treatment of cancers and other diseases [12]. In this way, I hope that my son continues to “pay it forward,” as we educate the next generation of scientists and engineers.

## Abbreviations

AMC: Albany Medical College
AML: acute myeloid leukemia
BEP: bleomycin, etoposide, cisplatin
CAR-T: chimeric antigen receptor
MSKCC: Memorial Sloan Kettering Cancer Center
VIP: etoposide, ifosfamide, cisplatin
